# Effectiveness of Non-Pharmacological Interventions in the Management of Pediatric Chronic Pain: A Systematic Review

**DOI:** 10.3390/children11121420

**Published:** 2024-11-25

**Authors:** Abel Checa-Peñalver, Cristina Lírio-Romero, Esther A. Luiz Ferreira, Sonsoles Hernandes-Iglesias, Inmaculada García-Valdivieso, Juan Manuel Pérez-Pozuelo, Sagrario Gómez-Cantarino

**Affiliations:** 1 Toledo University Hospital (HUT), Pediatric Hospitalization, Castilla-La Mancha Health Service (SESCAM), University of Castilla-La Mancha, Toledo Campus, 45071 Toledo, Spain; 2 Research Group of Pediatric and Neurologic Physiotherapy, ImproveLab, Universidad de Castilla-La Mancha, 45071 Toledo, Spain; cristina.lirio@uclm.es; 3 Department of Medicine, Federal University of Sao Carlos, Sao Carlos Campus, Sao Paulo 13565-905, Brazil; estherferreira@ufscar.br; 4 Health Sciences Faculty, Francisco de Vitoria University, M-515, km 1, 800, 28223 Pozuelo de Alarcón, Spain; s.hernandez@ufv.es (S.H.-I.); sagrario.gomez@uclm.es (S.G.-C.); 5 Faculty of Physiotherapy and Nursing, University of Castilla-La Mancha, 45071 Toledo, Spain; inmaculada.garciavaldivieso@alu.uclm.es (I.G.-V.); juanmanuel.perez5@alu.uclm.es (J.M.P.-P.); 6 Health Sciences Research Unit: Nursing (UICISA: E), Coimbra Nursing School (ESEnfC), 3004-011 Coimbra, Portugal

**Keywords:** pediatric chronic pain, non-pharmacological interventions, pain management, children, systematic review

## Abstract

(1) Background: Chronic pain in children remains an under-researched area, especially compared to acute pain. This systematic review aims to evaluate the effectiveness of non-pharmacological interventions in the management of pediatric chronic pain and their impact on the well-being of both children and their families. Given the growing interest in integrative treatments to reduce reliance on pharmacological solutions, this review addresses the need for alternative therapeutic approaches. (2) Methods: A systematic review was conducted following the PRISMA guidelines, covering studies published between 2019 and 2024 from PubMed, Cochrane Library, Web of Science, and Scopus. Eligible studies included children aged 2 to 18 years with chronic pain who received non-pharmacological interventions. Data were extracted on intervention types, participant characteristics, and outcomes. The risk of bias was assessed using RoB2 for randomized trials and ROBINS-I for non-randomized studies. (3) Results: A total of 11 studies involving 1739 children were included, assessing interventions such as cognitive behavioral therapy, personalized psychosocial follow-up programs, hypnotherapy, music therapy, and digital tools. The results demonstrated significant reductions in pain severity, improvements in emotional and physical well-being, and high patient satisfaction. However, the generalizability of findings was limited by the small sample sizes and variability in study designs. (4) Conclusions: Non-pharmacological interventions appear effective in managing pediatric chronic pain, offering improvements in pain reduction and quality of life. Further research is needed to optimize these approaches and confirm their long-term benefits across diverse populations. These interventions represent promising alternatives or complements to pharmacological treatments in pediatric pain management.

## 1. Introduction

Pain, according to the International Association for the Study of Pain (IASP), is characterized as “an unpleasant sensory and emotional experience associated with actual or potential tissue damage, or described in terms of such damage” [1]. Pain can also be divided into several categories as observed to define pain: duration, pathogenesis, course, and intensity [2].

In terms of duration, acute pain is characterized by its temporary onset in response to noxious stimuli, following a normal and predictable physiological response, while procedural pain refers to pain that arises as a result of diagnostic-therapeutic procedures. Chronic pain, on the other hand, is pain that persists beyond three months without a justifiable organic cause [3].

Although there is a wealth of research on acute pain management, especially in hospital emergency settings and surgical procedures, studies on chronic pain in children are comparatively scarce [4,5,6]. This disparity in research highlights the urgent need to focus more on chronic pain. While acute pain is generally well-understood and managed with established protocols, chronic pain requires more complex and personalized approaches due to its significant and multifaceted impact on the patient’s life [7,8]. Moreover, chronic pain can persist and profoundly affect various aspects of a child’s well-being, increasing the risk of developing long-term anxiety disorders and depression. Studies have shown that effective chronic pain management is associated with significant improvements in quality of life, emotional well-being, and social participation. Addressing chronic pain management is therefore essential not only to alleviate physical symptoms but also to improve the quality of life of this vulnerable population [9,10].

Some authors have pointed out that chronic pain is a recurrent issue in childhood. Approximately 30% of children and adolescents experience pain on a daily basis, and up to 35% of these have had pain for a period longer than 6 months. The most common causes of pain reported were headache (65.6%), abdominal pain (47.7%), limb pain (46.4%), and back pain (38.6%) [11,12].

The assessment and management of chronic pain in children is a challenge in itself due to the difficulty of verbalization and concerns about possible side effects of opioids, such as cognitive dysfunction, constipation, psychiatric comorbidities, and respiratory depression. Because of these opioid side effects, multidisciplinary analgesic treatments have increased. The simultaneous use of integrative pharmacological and non-pharmacological treatments may reduce the need for opioid treatment and improve the patient’s and family’s perception of well-being [13,14,15,16].

Evidence for the use of non-pharmacological methods of pain relief in pediatrics has been increasing in recent years [14,17,18]. These are classified as supportive (virtual reality, video games), cognitive (guided distraction, music), behavioral (breathing and relaxation techniques), and physical (control of environmental factors, application of heat and cold, etc.) [19].

In this context, the use of multidisciplinary interventions has proven to be a promising option. These interventions, which combine strategies such as pain education, sleep hygiene, school support, and cognitive behavioral therapy, among others, have shown significant improvements in pain intensity and functional disability in controlled studies [20]. Likewise, due to the recent COVID-19 pandemic that the world experienced, the possibility of experimenting with new therapies based on the use of web applications has also been explored [21,22].

Continued research in this field is essential to improve the understanding of the underlying mechanisms of chronic pain and to develop more effective and personalized approaches [23,24].

Therefore, this study aims to analyze the efficacy of non-pharmacological analgesia methods in the management of pediatric chronic pain, as well as the impact of these interventions on the child’s perception of well-being.

## 2. Materials and Methods

This study is compliant with the Preferred Reporting Items for Systematic Reviews and Meta-analyses (PRISMA) guidelines. A statement has been submitted for review and is currently pending acceptance. Additionally, this study has been registered in PROSPERO under the ID 613517.

The bibliographic search took place from 4 to 25 February 2024. The selection of non-pharmacological interventions in this review was based on their evidence and applicability in the context of pediatric chronic pain management. Interventions were chosen with a holistic approach, addressing both the physical and emotional aspects of chronic pain, considering their proven effectiveness and acceptability in the pediatric population.

A systematic search of PubMed, Cochrane Library, Web of Science, and Scopus was performed. To identify relevant studies, search terms combined using Boolean operators AND, OR, and NOT were used, allowing for the precise selection of articles that met the criteria established for this review. After examining the types of non-pharmacological treatment, the search strategy included the following terms: (child) AND (non-pharmacological) OR (non-pharmacological interventions OR (massage) OR (sensory stimulation) OR (imagination) OR (mind-body therapy) OR (psychological therapy) OR (art therapy) OR (hypnosis) OR (breathing technique) OR (biofeedback) OR (music therapy) OR (distraction) OR (relaxation training) OR (emotional therapy) AND (chronic pain). The full search strategy, including the specific terms and combinations used, can be found in File S1.

Studies were selected according to the following inclusion criteria, which were (a) children aged 2–18 years, without restriction of gender or ethnicity; (b) patients who maintained pain for more than 3 months; (c) the use of a non-pharmacological pain management intervention; (d) an assessment of outcomes using validated scales, such as the Numeric Rating Scale (NRS) or the Pediatric Quality of Life Inventory (PedsQL); and (e) studies published between 2019 and 2024.

On the other hand, exclusion criteria were studies (a) involving children without chronic pain, (b) not including non-pharmacological pediatric pain management interventions. The following data were extracted from the selected studies: (a) information on authors and year of publication; (b) country of intervention; (c) sample characteristics (sample size, age, and scales used); and (d) intervention characteristics.

Although several non-pharmacological interventions exist, such as pain neuroscience education, aerobic exercise, graded exposure therapy, and resilience training, these were not included in the present review due to a limited number of studies that met the established inclusion criteria, as well as limitations of applicability in pediatric populations due to cognitive or physical requirements.

The risk of bias in randomized controlled trials (RCTs) was assessed using the Cochrane RoB2 tool [25]. This tool analyzes five domains: the randomization process, deviations from intended interventions, missing data on outcomes, measurement of outcomes, and selection of reported outcomes. A study was considered “low risk” if it scored “low risk” in all domains, “some concerns” if any domain was rated as “some concerns”, and “high risk” if at least one domain was assessed as “high risk” or several domains were rated with “some concerns”.

On the other hand, the risk of bias in non-randomized clinical trials was assessed using the ROBINS-I tool [26], which includes seven domains: confounding, participant selection, classification of interventions, deviations from intended interventions, missing data on outcomes, outcome measurement, and selection of reported outcomes. Bias was classified as “low risk” if the study was assessed with low scores in all domains, “moderate risk” if the rating was moderate in all domains, “serious risk” if at least one domain was considered serious, and “critical risk” if at least one domain was assessed as critical.

The risk of bias assessment was performed independently by at least two investigators (A.C.P. and S.G-C), who analyzed each study using the corresponding tools (RoB2 for randomized studies and ROBINS-I for non-randomized studies). In the case of discrepancies between the assessors, a third investigator (E.A.L.F) reviewed the study and provided an unbiased assessment, which was used to reach a final consensus.

In the data selection and collection process, three authors (A.C.P., S.G.-C., and E.A.L.F.) independently reviewed each publication for compliance with the stated inclusion criteria and then jointly made decisions about which data to include in the review. A 4th author carried out a review when there was discord among the three reviewers (S.H).

## 3. Results

**Figure 1:**
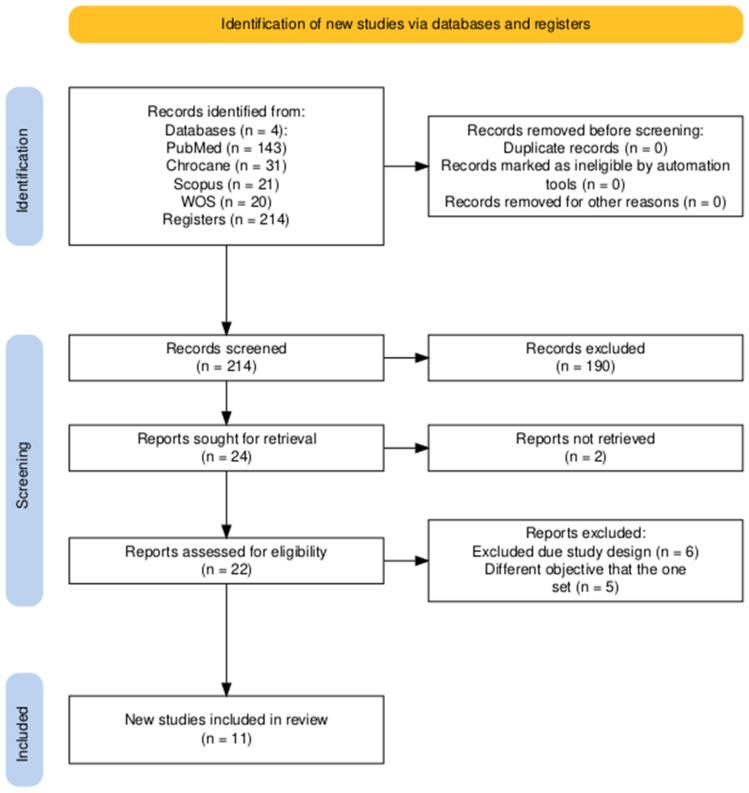
Flowchart for study selection. (Notes on the figure. WOS: Web of Science.).

The search identified 11 studies (Figure 1) [27,28,29,30,31,32,33,34,35,36,37] that were included in this systematic review. The total number of children studied was (*n* = 1739), the total number of cases (individuals who were part of an intervention) was (*n* = 924), and the total number of controls (individuals who were part of treatment as usual) was (*n* = 815). The minimum number of children in a study was (*n* = 2) [36], while the maximum number of individuals in a study was (*n* = 419) [28]. The minimum age was 2 years [30], the maximum was 18 years, and the mean age across all studies was 11.92 years (Figure 1) (Table 1). All articles were passed by the appropriate ethics committee, ensuring compliance with ethical standards in pediatric research (File S2).

For the measurement of chronic pediatric pain, five studies used the Numerical Rating Scale (NRS) [28,31,34,36,37], i.e., 45.54%, while the remaining studies used other scales, such as the Visual Analog Scale (VAS) [28], Chronic Pain Grading (CPG) [28], PROMIS Paediatric Pain Interference-Short [32,36], and the Abdominal Pain Index (API) [32]. In the studies that did not use a scale to measure pediatric chronic pain, scales were used to measure well-being in pediatric patients with chronic pain, such as the Pediatric Quality of Life Inventory (PedsQL) [37], Depression Anxiety and Stress Scales (DASS-21) [29], and the Child Activity Limitations Interview (CALI-9) [28,34], the latter being the only one used in more than one study (18.18%). As for the procedures performed in the intervention group for the management of chronic pain, a study explored hypnotherapy [27], another explored music therapy [30], four studies explored face-to-face and digital cognitive behavioral therapy (CBT) [29,32,34,37], three studies explored psychosocial therapies and psychological support [28,31,33], and two studies conducted technology-based interventions and digital therapies using web and smartphone applications [35,36].

### 3.1. Risk of Bias in the Studies

**Figure 2:**
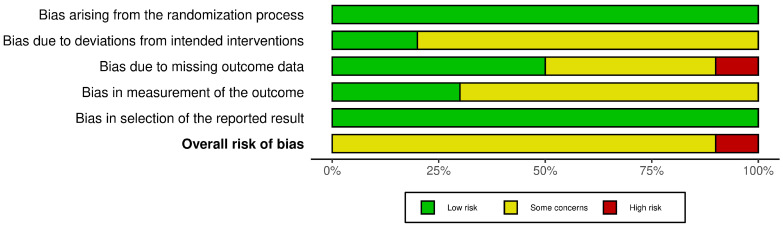
Risk of bias assessment in randomized controlled trials (RoB 2) [31].

**Figure 3:**
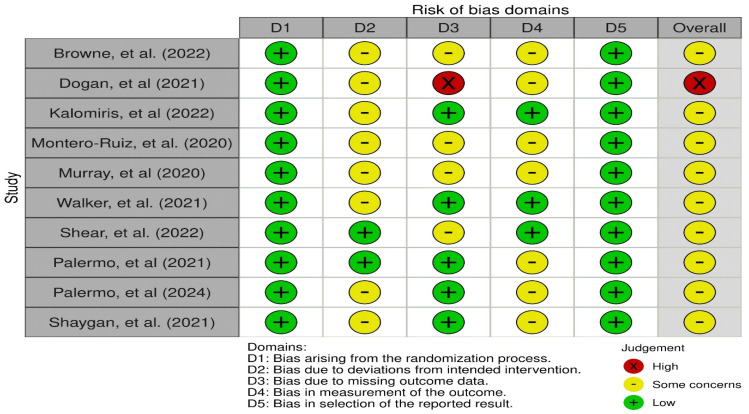
Risk of bias assessment for each included study [27,28,29,30,31,32,33,34,35,37].

According to the assessment of the risk of bias in randomized clinical trials (RCTs) using the RoB2 method, 9.09% of the studies [28] showed a high risk of bias, while 90.91% [27,29,30,31,32,33,34,35,36] showed a moderate risk of bias. Specifically, 100% of the studies had a low risk of bias in the randomization process and selection of the reported outcome, 18.18% [34,35] had a low risk of bias in the deviation from planned interventions, 9.09% [28] had a high risk of bias in missing outcome data, and 63.64% [28,29,31,32,35,36,37] had a moderate risk of bias in the measurement of the outcome (Figure 2). The assessment of each study is shown below (Figure 3).

**Figure 4:**
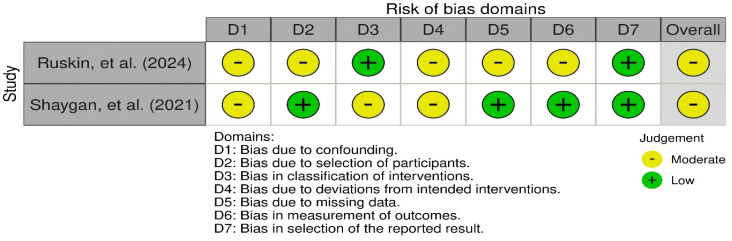
Risk of bias assessment in quasi-experimental studies (ROBINS-I) [36,37].

In the non-randomized trials, a risk of bias assessment with the ROBINS-I scale revealed that 100% of the studies had a moderate risk of bias. Specifically, 50% of the studies [37] reflected a low risk of bias in participant selection, bias in the classification of interventions, bias due to missing data, and bias in measurement. A total of 100% reflected a low risk of bias in the selection of the reported result and a moderate risk of bias due to confounding and bias due to deviations from intended interventions (Figure 4).

### 3.2. Effectiveness of Interventions

The article on hypnotherapy [27] focused on evaluating the effectiveness of this intervention in reducing the severity of nausea in pediatric patients with chronic pain, reporting a 45% reduction in nausea severity and statistically significant improvements (*p* < 0.05). On the other hand, Montero-Ruiz, in his work on music therapy [30], documented a marked improvement in time perception and respiratory function among children who received music therapy. Children who received music therapy showed a 30% increase in their perception of enjoyment during respiratory physiotherapy and a 25% reduction in the perceived time of the session, with significant improvement in lung function (*p* = 0.01).

In relation to the effectiveness of the personalized psychosocial follow-up program (PAC), the findings were mixed; Shear [33] demonstrated that a combined physical and psychological therapy approach significantly reduced pain severity, as measured by the Numerical Rating Scale (NRS) and Visual Analog Scale (VAS). The reduction in pain severity across the intervention group was consistent, with average NRS scores decreasing by approximately 2.1 points after treatment (*p* < 0.01). Similarly, the study by Dogan [28] showed that the PAC group experienced a significant reduction in pain intensity, dropping from 8.23 to 6.33 on the NRS at 6 months (*p* < 0.01), when compared to the group that did not receive aftercare. Kalomiris [29] found that the psychosocial program led to a 15% improvement in lung function, with a significant increase in vital capacity (*p* = 0.02) in children who participated in regular physical activity as part of the intervention.

Regarding the impact of cognitive behavioral therapy (CBT), whether face-to-face or digital, the following results were obtained: Palermo (2020) [34] evaluated a digital CBT program on chronic pain-related disability in pediatric patients, and although the overall effects were not significant (*p* > 0.05), it was found that greater engagement with the program correlated with better outcomes (*p* < 0.01). Shaygan [37] reported that group CBT led to significant reductions in chronic pain intensity, with patients reporting a reduction from 7.8 to 5.4 on the NRS scale after 8 weeks of therapy (*p* < 0.001). This result is in line with the study by Walker [32], in which CBT improved coping strategies and reduced pain interference, with patients showing a reduction in pain interference from 6.5 to 4.1 on a scale of 0–10 after 6 months of therapy (*p* = 0.001). Finally, the work of Murray [31] showed that online CBT significantly reduced pain-related disability, with participants reporting a 30% improvement in daily functioning (*p* < 0.01), and this improvement was sustained for 12 months.

Finally, on interventions based on new technologies, Ruskin [36] used virtual reality and game therapy, which showed a significant reduction in anxiety in children with chronic pain, with anxiety scores dropping by 25% (*p* = 0.02) after 4 weeks of therapy. Another study by Palermo (2024) [35] also showed that CBT significantly improved fatigue and pain interference, with fatigue scores decreasing by 20% (*p* = 0.01) and pain interference reduced by 18% (*p* < 0.01).

A comparative summary of the main findings is presented below, detailing the results obtained, the sustainability of the effects, adherence and accessibility of each intervention, together with the associated statistical significance values (Table 2).

## 4. Discussion

This systematic review examines the effects of non-pharmacological analgesia methods in reducing chronic pain in pediatric patients. The results show that interventions such as hypnotherapy, music therapy, cognitive behavioral therapy (CBT), psychosocial care therapies (PAC), psychosocial therapies, and new technology-based interventions can be effective in reducing pain-related parameters, anxiety, quality of life, physical functioning, and pain catastrophizing.

Cognitive behavioral therapies (CBT) [31,32,34,37], in both face-to-face and Internet-based formats, were consistently effective in reducing pain intensity and pain-related disability, especially in specific subgroups, such as those with dysfunctional psychological profiles. For example, adolescents who participated in CBT programs showed a significant decrease in pain scores. For example, in Murray’s study [31], the reduction in pain was 2.4 points on the NRS, (*p* < 0.01). These findings are consistent with previous work [38,39,40,41] that stated that when CBT is adapted for chronically ill adolescents, it is effective in reducing pain and improving psychological well-being. These adaptations include customization according to patient profile, the use of cognitive restructuring techniques and behavioral activation, and family involvement, which reinforces the efficacy of CBT in complex contexts of chronic pain and comorbidities.

In turn, the personalized psychosocial follow-up programs (PACs) demonstrated a significant reduction in pain severity, and improvements in emotional well-being were reported [28]. Also, the reduction of anxiety in caregivers and the positive correlation with pain reduction in patients was highlighted [28], showing a 25% reduction in anxiety scores (*p* < 0.01) and improvements in quality of life. These results are consistent with other published work [42,43] indicating that it can contribute to improving the emotional and functional well-being of patients, helping them to develop more effective coping strategies, reduce anxiety and depression, and improve their quality of life.

Hypnotherapy [27] was effective in reducing pain symptoms in 76.6% of participants (*p* < 0.05), with significant improvements in pain perception and emotional well-being. Specifically, participants receiving hypnotherapy showed a reduction in pain scores by an average of 2.5 points on the pain scale compared to a reduction of 1.2 points in the SMT group, indicating a significant difference (*p* < 0.05).

Music therapy [30] reported a significant reduction in pain intensity of 20% (*p* = 0.01) and improvements in emotional well-being, as well as a reduced perception of the duration of interventions. These findings were consistent with previous studies on music therapy in the pediatric population [44,45]. As with hypnotherapy, the analysis was conducted with a relatively small sample size, which limits the generalizability of the results to a larger population.

Finally, the reviewed studies on new technology-based interventions [35,36] reported positive results, with an intervention delivered via a mobile application resulting in a significant reduction in daily pain and improvements in quality of life. Another study evaluating the effectiveness of a pain management program through an interactive digital platform found a significant decrease in pain intensity (and improvements in participants’ physical and emotional functioning) [36]. These results were consistent with other studies that also evaluated new technologies for pain management in the pediatric population [46,47], underlining their potential as valuable tools in the treatment of pediatric chronic pain, providing accessible and effective options to improve patients’ well-being.

Adherence to the interventions was high overall, with rates ranging from 70% to 85%. Participants found the interventions accessible and easy to integrate into their daily routines [36,37]. In the case of papers that addressed digital interventions [35,36], flexibility and ease of use at any time and place were identified as determining factors. In the case of CBT interventions, the effectiveness perceived by patients and the constant support and supervision by therapists could explain the high adherence rate [29,31]. Interventions that used hypnotherapy [27] and music therapy [30] also had high adherence, between 80% and 90%, due to their easy integration with other treatments.

Participants expressed a high level of satisfaction with the digital interventions, highlighting the ease of use and relevance of the content for pain management. In the case of digital interventions and CBT, their interactivity and personalization are highlighted, as well as the immediate feedback and sense of progress experienced by children [31,32,34,37]. For hypnotherapy-based interventions [27] and music therapy [30], a high satisfaction rate (80–92%) was also observed due to the subjectively positive and relaxing experience that patients reported.

### Limitations of This Study

Despite promising results, there are certain limitations associated with these non-pharmacological interventions. For CBT, while it is safe and effective, limitations exist concerning the duration of its effects, highlighting the need for further research to optimize its implementation, explore therapeutic combinations, and develop strategies to ensure sustained long-term benefits [37,38,39,40,41]. For PAC programs, more research is needed to determine the optimal conditions for implementation, such as the frequency and duration of sessions and their combination with other treatment modalities, to maximize long-term benefits [28,29,42,43]. The studies on hypnotherapy and music therapy had relatively small sample sizes and lacked prolonged follow-up, limiting the generalizability of their results to larger populations. Robust research is needed to validate the effectiveness and sustainability of these therapies in the long-term management of pediatric chronic pain [44,45,48,49]. Similarly, the studies on new technology-based interventions also had small sample sizes, and further research is required to confirm their effectiveness and sustainability in broader contexts. The inherent limitations of this review include a high risk of bias in the articles considered. To improve the external validity of the results, larger sample sizes and more specific age ranges are necessary. Additionally, clinical heterogeneity due to differences in the pain measurement scales, types of intervention, and control groups could impact the study outcomes [35,46,47].

The search was limited to studies from 2019 to 2024 to ensure relevance, capturing the latest advancements in non-pharmacological pain management, including digital and mobile-based interventions. This timeframe reflects current practices and methodologies, enhancing the applicability of findings. However, this focus may introduce temporal bias by excluding older studies with established long-term efficacy, potentially affecting the representativeness of evidence. While recent studies provide insights into short- and medium-term outcomes, a broader range of publication dates might offer a more comprehensive view of intervention effectiveness over time.

A key limitation of this systematic review is the substantial heterogeneity among the included studies. Variations in intervention types, ranging from CBT and hypnotherapy to music therapy and digital health applications, present challenges in directly comparing outcomes. Each intervention type employs unique mechanisms, which may yield different effects on pain and well-being, thus complicating efforts to generalize findings across interventions.

Additionally, studies utilized diverse outcome measurement scales, including the NRS, API, and FDI, each capturing pain intensity and functional improvement differently. This diversity in measurement tools limits the ability to aggregate data consistently and may introduce variability in interpreting the effectiveness of interventions. While these differences reflect the individualized nature of non-pharmacological pain management, they also emphasize the need for standardized outcome measures to enhance comparability in future research.

## 5. Conclusions

Non-pharmacological therapies represent valuable alternatives to or complement traditional pain management, reducing dependence on medications such as opioids, which carry risks of significant side effects. Interventions such as cognitive behavioral therapy (CBT), music therapy, and hypnotherapy not only help to alleviate pain intensity but also promote improved quality of life, emotional well-being, and social engagement, potentially mitigating the risk of long-term psychological problems such as anxiety and depression. The accessibility and ease of integration of many of these interventions, especially technology-based options, such as mobile apps, contribute to high patient adherence and satisfaction, offering interactive and engaging ways for children to manage their pain. In addition, the involvement of family members in the treatment process, particularly through approaches such as CBT, improves treatment outcomes and reduces caregiver stress, reinforcing a family-centered model of care. The adaptability of technology-based interventions also allows for broad implementation in a variety of settings, including hospitals, clinics, and home environments, making these options accessible even in areas with limited specialized resources. Finally, the ability to personalize these treatments to each child’s unique psychological and pain profiles underscores their value in providing personalized care, ultimately promoting better coping strategies and improving functionality.

This systematic review underlines the importance of adopting a multimodal approach to preventing pediatric pain and seeking alternatives to pharmacological treatment. Both for health professionals and parents, who are the main caregivers. This implies the need to implement training, observation, and the assessment of pain in children. In this field of research, this review highlights the existence of gaps in current knowledge and the urgent need for further studies to assess the safety and efficacy of the proposed interventions. It is also recommended that future research should examine the potential long-term consequences of untreated pediatric pain.

This study allows us to conclude that the non-pharmacological techniques analyzed could be effective in reducing chronic pain in the pediatric population. The results showed an improvement in terms of pain intensity, quality of life, and emotional, physical, and social well-being of children and their environment.

In summary, although these techniques show great potential as alternatives or complements to pharmacological treatments, further research is crucial to optimize their application and better understand their impact on various aspects of pediatric chronic pain.

## Figures and Tables

**Table 1:** Characteristics of included studies.

Authors and Country	PA Measurement and Method	Sample Characteristics	Age and Sex	Main Variables	Intervention Duration	Outcomes
Browne, et al. (2022) [27]. The Netherlands	IC: hypnotherapy CG: standard medical care	Patients: (n = 100) IC: (n = 50) (n = 3) Excluded CG: (n = 50) (n = 3) Excluded	Age: 8–18 yrs. Mean age of IC: 14.9 yrs. Mean age of CG: 14.5 yrs. Gender: IC: (n = 36) F, (n = 11) M. CG: (n = 33) F, (n = 14) M.	Symptoms: duration, severity, and incidence. Self-perception. Physical and psychological well-being.	3 months, with follow-up lasting up to 12 months post-intervention	Nausea Severity Scale, Nausea Incidence Scale, Nausea Frequency Scale
Dogan, et al. (2021) [28]. Germany	IC: PAC CG: standard medical care	Patients: (n = 419) IC: (n = 201) (n = 86) Excluded. CG: (n = 218) (n = 111) Excluded	Age: 8–17 yrs. Mean age of IC: 14.3 yrs. Mean age of CG: 14.3 yrs. Gender: IC: (n = 148) F, (n = 53) M. CG: (n = 155) F, (n = 63) M	Pain duration. Pain location. Pain intensity. School absenteeism	6 months. The follow-up period was 6 months	Chronic Pain Grading, NRS, Revised Child Anxiety and Depression Scale
Kalomiris, et al. (2022) [29]. USA	IC: CBT, CG: standard medical care	Patients (n = 89) and Caregivers. IC: (n = 44) (n = 4) Excluded. CG: (n = 45) (n = 6) Excluded	Age: 9–14 yrs. Mean age of IC: 11.67 yrs. Mean age of CG: 11.74 yrs. Gender: IC: (n = 20) F, (n = 20) M. CG: (n = 27) F, (n = 12) M	Primary diagnosis. Participating caregiver. Child symptoms at baseline. Caregiver symptoms at baseline	The intervention and follow-up were conducted together during the 8 weeks of the study	Depression Anxiety and Stress Scales, Functional Disability Inventory, Visual Analog Scale
Montero-Ruiz, et al. (2020) [30]. Spain	IC: music therapy GC: standard medical care. PG: music chosen by the patients	Patients: (n = 54) IC: (n = 18) (n = 3) Excluded CG: (n = 18) (n = 3) Excluded PG: (n = 18) (n = 5) Excluded	Age: 2–17 yrs. Median age: CG: 7.2 ± 4.3 (2–16) PG: 8.1 ± 5.6 (2–17) IC: 7.9 ± 4.7 (2–17) Gender (M): CG: 8 (53.3%) PG: 9 (69.2%) IC: 10 (66.7%)	Time to perform an intervention. Enjoy your thoracic physiotherapy routine. Avoided use of health resources	3 months, with a follow-up period of 3 months	7-point Likert scales, Measurement of time perception
Murray, et al. (2020) [31]. USA	IC: CBT CG: standard medical care	Patients (n = 273) and Caregivers. IC: (n = 138) (n = 4) Excluded CG: (n = 135)	Age: 11–17 yrs. Mean age: IC: 14.64 (1.62) CG: 14.70 (1.72) Gender (% F): IC: 78.3% CG: 71.9%	Ethnicity Primary pain location Parental education Household annual income	8 and 10 weeks, with a structured follow-up period at 6 months and at 12 months	NRS, CALI, BAPQ
Walker LS, et al. (2021) [32]. USA	IC: PAC CG: standard medical care	Patients: (n = 300) IC: (n = 148) (n = 12) Excluded CG: (n = 152) (n = 10) Excluded	Age: 11–17 yrs. Mean age: IC: 14.62 (1.88) CG: Not specified Gender: IC: 66.2% F CG: Not specified	Symptom characteristics Ethnicity Parental employment and education Family member who participated	8 weeks, with a follow-up period of 12 months after the intervention	CSSI-24, Abdominal Pain Index, PROMIS Pediatric Pain Interference-Short Form 8a
Shear D, et al. (2022) [33]. USA	IC: PAC CG: Standard care	Patients: (n = 68) IC (n = 36) (n = 2) Excluded CG: (n = 32) (n = 4) Excluded	Age: 8–18 yrs. Mean age: IC: 14.2 yrs CG: Not specified Gender: IC: (n = 29) F, (n = 7) M CG: (n = 26) F, (n = 6) M	Pain-related fear and avoidance Functional disability	8 weeks, with a follow-up period of 6 months after the intervention ended	Fear of Pain Questionnaire. Functional Disability Inventory
Palermo TM; et al. (2020) [34]. USA	IC: internet CBT CG: standard care	Patients: (n = 143) IC: (n = 73) (n = 19) Excluded. CG: (n = 70)	Age: 10–17 yrs. Mean age: IC: 14.4 yrs CG: 14.6 yrs Gender: IC: (n = 58) F, (n = 15) M CG: (n = 59) F, (n = 11) M	Pain-related disability, pain intensity	8 weeks for the INT, followed by a 3-month follow-up after the completion of the treatment	CALI-9, NRS, PGIC
Palermo TM; et al. (2024) [35]. USA	IC: internet CBT CG: standard care	Patients: (n = 111) IC: (n = 57) (n = 8) Excluded CG: (n = 54) (n = 5) Excluded	Age: 12–18 yrs. Mean age: IC: 14.9 yrs CG: 14.8 yrs Gender: IC: (n = 34) F, (n = 23) M CG: (n = 31) F, (n = 23) M	Pain intensity, Number of days with pain, activity limitations	12 weeks. A follow-up was conducted at 6 months to assess long-term outcomes	CALI-9, Coping Strategies Questionnaire for Sickle Cell Disease
Qualitative Studies						
Ruskin D, et al. (2024) [36]. USA	IC: technology-based interventions	Patients: (n = 2) and their caregivers	Age: 8–18 years Mean age IC: 14.5 years	Chronic postsurgical pain Pain catastrophizing Pain interference with daily life	12 months after the intervention	NRS, PROMIS, Pain Catastrophizing Scale
Mixed studies						
Shaygan M, et al. (2021) [37]. Iran	IC: Internet CBT CB: standard care	Quantitative: Patients: (n = 128) IC: (n = 64) (n = 14) Excluded. CG: (n = 64) Qualitative: Patients: (n = 14)	Quantitative: Age: 12–19 yrs. Mean age: IC: 13.81 ± 1.72 CG: 13.65 ± 1.72 Gender: IC: 38 (55.1%) F CG: 31 (44.9%) F Qualitative: Age: 12–17 yrs. Gender: (n = 9) F (n = 5) M	Quantitative: Pain intensity Quality of life Mother’s and father’s education and occupation Qualitative: Physical and psychological pain management Interpersonal resources	4 weeks. A follow-up was conducted 3 months later	NRS, Pediatric Quality of Life Inventory, CSQ-I

Notes on the table: IC: intervention group, CG: control group, M: male, F: female, PA Measurement and Method: pain assessment measurement and method, Intervention Duration: duration of the intervention and measurement period, Outcomes: tool used for measuring and evaluating the result. PAC: psychosocial aftercare program, CBT: cognitive behavioral therapy, CALI: Child Activity Limitations Interview, BAPQ: Bath Adolescent Pain Questionnaire, CSSI-24: Children’s Somatic Symptoms Inventory-24, PGIC: Patient Global Impression of Change, CALI-9: Child Activity Limitations Interview-9. NRS: Numeric Rating Scale. PROMIS: Patient-Reported Outcomes Measurement Information System. CSQ-I: Client Satisfaction Questionnaire adapted to Internet-based Interventions.

**Table 2:** Comparison of the effectiveness of interventions.

Intervention	Description of the Intervention	Main Outcomes	Sustainability of Effects	Adherence and Accessibility	*p* Value
Hypnotherapy [27]	Evaluation of hypnotherapy to reduce nausea severity in pediatric chronic pain.	45% reduction in nausea severity.	Effects sustained up to 12 months.	Requires access to specialized hypnotherapy; high reported adherence.	*p* < 0.05
Music Therapy [30]	Evaluation of music therapy to improve chronic pain during physiotherapy sessions	Increased adherence to physiotherapy (80% vs. 60% in control group); significant improvement in lung function.	Not specified;	High accessibility; applicable at home; good adherence.	*p* = 0.01
PAC [28]	Psychosocial follow-up program after intensive treatment for pediatric chronic pain.	Reduction in pain on NRS from 8.23 to 6.33 after 6 months.	Effects sustainable up to 6 months post-discharge.	High accessibility in hospitals and clinics; adherence improved with continuous social support.	*p* < 0.01
PAC [31]	Internet PAC to reduce disability from chronic pain.	30% reduction in disability and improvement in daily functioning over 12 months.	Effects sustainable for up to 12 months.	High digital accessibility; moderate adherence with 65% completion rates.	*p* < 0.01
PAC [33]	Combination of physical and psychological therapy in PAC.	Average pain reduction of 2.1 points on NRS.	Equivalent effects between virtual and in-person treatment; short-term follow-up.	Increased enrollment due to virtual modality; adherence like in-person format.	*p* < 0.01
CBT [29]	CBT for functional abdominal pain.	FDI scores reduced from 17.87 to 11.33, VAS scores from 3.86 to 2.50, and anxiety (SCARED) scores from 35.12 to 25.46.	Improvements observed post-treatment; no long-term follow-up data.	Brief and easy-to-implement intervention; high adherence during the intervention.	*p* < 0.01
CBT [32]	Online CBT to reduce pain interference.	Pain interference reduced from 6.5 to 4.1 on a 0–10 scale.	Effects maintained for up to 12 months in HPD subgroup; less pronounced effects in other subgroups.	Good adherence to online platform; high accessibility for participants.	*p* = 0.001
CBT [34]	Digital CBT to improve pain-related disability.	Better outcomes with higher engagement in the program.	Significant effects on pain up to 6 months.	High accessibility in clinics and community; variable adherence among users.	*p* < 0.01 (for correlation)
CBT [37]	Group CBT for chronic pain.	Reduction in NRS from 7.8 to 5.4 after 8 weeks.	Effects maintained up to 3 months follow-up.	High adherence (78.12%); high accessibility due to smartphone use.	*p* < 0.001
Technology-Based Interventions [35]	Digital CBT for adolescents with sickle cell disease.	Fatigue reduced by 20% and pain interference by 18%.	Significant effects on pain and fatigue for up to 6 months.	High accessibility in clinics and community; variable adherence among users due to engagement levels.	*p* = 0.01 (fatigue), *p* < 0.01 (pain)
Technology-Based Interventions [36]	Virtual reality and games to reduce anxiety in children with chronic pain.	25% reduction in anxiety levels after 4 weeks of therapy.	Preliminary results; no long-term data; need for additional follow-up.	Facilitate implementation in hospital programs; high adherence during the preoperative period.	*p* = 0.02

## Supplementary Materials

The following supporting information can be downloaded at: https://www.mdpi.com/article/10.3390/children11121420/s1, File S1. Search Strategy; File S2. Table of Studies and Ethical Approval (Including All 11 Studies).
