# Supplementary material for: Effectiveness of Non-Pharmacological Interventions in the Management of Pediatric Chronic Pain: A Systematic Review
Source: Children (Basel). 2024 Nov 25;11(12):1420. doi: 10.3390/children11121420 (PMC11674135; doi:10.3390/children11121420)
Supplement: Supplementary file 1 [file children-11-01420-s001.zip › children-3282975-File S1 Children.pdf]

File S1. Search Strategy.

Database	Search Syntax	Filters	Citations found
PUBMED	(((((((((((((((child) AND (chronic pain))) AND (non-pharmacological[Title/Abstract])) OR (non-pharmacological interventions[Title/Abstract])) OR (massage[Title/Abstract])) OR (sensory stimulation[Title/Abstract])) OR (imagination[Title/Abstract])) OR (mind-body therapy[Title/Abstract])) OR (psychological therapy[Title/Abstract])) OR (art therapy[Title/Abstract])) OR (hypnosis[Title/Abstract])) OR (breathing technique[Title/Abstract])) OR (biofeedback[Title/Abstract])) OR (music therapy[Title/Abstract])) OR (distraction[Title/Abstract])) OR (relaxation training[Title/Abstract])) OR (emotional therapy[Title/Abstract])	Free full text, Randomized Controlled Trial, in the last 5 years, English, Spanish, Child: birth-18 years	143
CHROCANE	(Infant OR child) AND Chronic Pain AND Non-Pharmacological Interventions NOT adult<br><br>PICO: 'Population ("Infant" OR "Child" OR "Chronic Pain") AND Intervention "Non-Pharmacological Interventions" AND Comparison "Usual Care" AND Outcome ("Pain" OR "Pain Relief")'	Free full text, 5 years (2019-2024), English, Spanish.	31

SCOPUS	(TITLE-ABS-KEY ("chronic pain") AND TITLE-ABS-KEY ("children" OR "child") AND TITLE-ABS-KEY ("non-pharmacological" OR "non-pharmacological interventions" OR "non-pharmacologic" OR "massage" OR "sensory stimulation" OR "imagination" OR "mind-body therapy" OR "psychological therapy" OR "art therapy" OR "hypnosis" OR "breathing technique" OR "biofeedback" OR "music therapy" OR "distraction" OR "relaxation training" OR "emotional therapy"))	Free full text, 5 years, English, Spanish.	21
WOS	((TS=(chronic pain)) AND TS=(children OR child)) AND TS=(non-pharmacological OR non-pharmacological interventions OR non-pharmacologic OR massage OR sensory stimulation OR imagination OR mind-body therapy OR psychological therapy OR art therapy OR hypnosis OR breathing technique OR biofeedback OR music therapy OR distraction OR relaxation training OR emotional therapy)	Free full text, 5 years (2024-2019), English, Spanish, clinical trial	20
