# Supplementary material for: Effectiveness of Non-Pharmacological Interventions in the Management of Pediatric Chronic Pain: A Systematic Review
Source: Children (Basel). 2024 Nov 25;11(12):1420. doi: 10.3390/children11121420 (PMC11674135; doi:10.3390/children11121420)
Supplement: Supplementary file 1 [file children-11-01420-s001.zip › children-3282975-File S2 children-11.25.pdf]

Table of Studies and Ethical Approval (Including All 11 Studies)

Article Title	Ethics Committee Approval
Skills or Pills: Randomized Trial Comparing Hypnotherapy to Medical Treatment in Children With Functional Nausea	Approved by the Ethics Committee of St. Antonius Hospital
Effectiveness of a Psychosocial Aftercare Program for Youth Aged 8 to 17 Years With Severe Chronic Pain	Trial registered in DRKS00015230,
Child-Focused Cognitive Behavioral Therapy for Pediatric Abdominal Pain Disorders Reduces Caregiver Anxiety	Registered in ClinicalTrials.gov: NCT03134950
Effects of Music Therapy as an Adjunct to Chest Physiotherapy in Children with Cystic Fibrosis	Approved by the Human Research Ethics Committee of the Regional Hospital of Málaga
Moderators of Internet-Delivered Cognitive-Behavioral Therapy for Adolescents With Chronic Pain	Approved by the Institutional Review Board at the primary site, Seattle Children's Research Institute,
A digital health psychological intervention (WebMAPMobile) for children and adolescents with chronic pain	Approved by the Seattle Children’s Research Institute and corresponding centers
A cognitive–behavioral digital health intervention for sickle cell disease pain in adolescents	Approved by ethics boards at participating institutions, including Seattle Children’s Hospital
Innovative Program to Prevent Pediatric Chronic Postsurgical Pain: Patient Partner Feedback on Intervention Development	Approved by the Research Ethics Board of the Hospital for Sick Children, REB #1000075212
The effect of a smartphone-based pain management application on pain intensity and quality of life in adolescents with chronic pain	Approved by the Ethics Committee of Shiraz University of Medical Sciences, IR.SUMS.REC.1398.1099
Rapid Transition to Virtual Assessment and Treatment in an Interdisciplinary Randomized Clinical Trial for Youth With Chronic Pain	Approved by the Institutional Review Board at Boston Children’s Hospital
Internet-delivered cognitive behavioral therapy for youth with functional abdominal pain	Approved by the Institutional Review Board of Vanderbilt University Medical Center
